# Broadband Wide Angle Lens Implemented with Dielectric Metamaterials

**DOI:** 10.3390/s110807982

**Published:** 2011-08-12

**Authors:** John Hunt, Nathan Kundtz, Nathan Landy, Vinh Nguyen, Tim Perram, Anthony Starr, David R. Smith

**Affiliations:** 1 Center for Metamaterials and Integrated Plasmonics, Department of Electrical and Computer Engineering, Duke University, Durham, NC 27708, USA; E-Mails: nkundtz@intven.com (N.K.); nathan.landy@duke.edu (N.L.); vinh.nguyen@duke.edu (V.N.); drsmith@ee.duke.edu (D.R.S.); 2 SensorMetrix, San Diego, CA 92121, USA; E-Mails: tperram@sensormetrix.com (T.P.); astarr@sensormetrix.com (A.S.)

**Keywords:** metamaterials, transformation optics, gradient index, Luneburg lens

## Abstract

The Luneburg lens is a powerful imaging device, exhibiting aberration free focusing for parallel rays incident from any direction. However, its advantages are offset by a focal surface that is spherical and thus difficult to integrate with standard planar detector and emitter arrays. Using the recently developed technique of transformation optics, it is possible to transform the curved focal surface to a flat plane while maintaining the perfect focusing behavior of the Luneburg over a wide field of view. Here we apply these techniques to a lesser-known refractive Luneburg lens and implement the design with a metamaterial composed of a semi-crystalline distribution of holes drilled in a dielectric. In addition, we investigate the aberrations introduced by various approximations made in the implementation of the lens. The resulting design approach has improved mechanical strength with small aberrations and is ideally suited to implementation at infrared and visible wavelengths.

## 1. Introduction

A Luneburg lens is a gradient index (GRIN) lens with a spherically or cylindrically symmetric index distribution that exhibits aberration-free focusing of light incident from any direction onto a spherical or cylindrical surface [1,2]. Despite its advantageous imaging properties, however, the Luneburg is not widely used since its focal surface is not compatible with standard detector and transmitter arrays. The unique properties of a Luneburg lens or similar optical element can be accessed utilizing a detector array built on a nonplanar surface that conforms to the focal surface of the device [3]. Alternatively, the possibility exists to seek a gradient index profile that retains the nearly ideal optical properties of the Luneburg lens, but allows one or more surfaces to be planar. Such a design was suggested in the context of the emerging field of transformation optics, where it was shown that a simple analytic coordinate transformation could be applied to flatten one side of the lens [4]. While the initially suggested transformation required a complex, anisotropic medium, Kundtz et al. have investigated the possibility of obtaining numerical transformations that, at least for cylindrical lenses with transverse electric (TE) polarization, result in a Luneburg lens with a flattened focal plane that can be implemented using only isotropic GRIN media [5]. The resulting lens retains nearly identical optical characteristics as the untransformed lens but focuses onto a flat surface over a limited field of view.

In the transformation optical design methodology, a desired distortion of the space that an electromagnetic wave travels through is achieved by introducing a spatial variation of the electric permittivity and magnetic permeability tensors over some region [6]. The resulting constitutive parameters associated with the transformation are in general anisotropic and inhomogeneous. However, conformal and quasi-conformal coordinate transformations preserve, either exactly or approximately, respectively, the orthogonality of space. In two dimensions, these transformations minimize the anisotropy of the prescribed index distribution allowing the anisotropy to be neglected so that the transformation may be implemented with only isotropic materials [7]. In three dimensions, however, anisotropy is always required [8–10]. For TE guided waves the permeability in the direction of the magnetic field remains equal to unity after a transformation is applied, thus the quasi-conformal transformation can be implemented with a variation in the isotropic permittivity, or index of refraction, alone.

In the flattened Luneburg lens reported by Kundtz *et al.*, the necessary isotropic permittivity distribution was implemented using an artificially structured metamaterial comprising non-resonant electric dipoles of varying size. This method of implementation may not scale well towards infrared (IR) and visible wavelengths, however, since metals behave less as conductors and increasingly like lossy dielectrics. Thus, for better scalability, a dielectric-only implementation would be preferable. In addition, it would be advantageous for the entire device to be fabricated from a single dielectric slab using routinely available lithographic techniques. A simple way of achieving these requirements is to drill uniformly sized sub-wavelength diameter holes in a dielectric slab such that the density of holes in a given location yields the desired index at that location. This approach has been used to fabricate a variety of quasi-conformal transformation optics devices, such as “carpet cloaks”, that operate at IR wavelengths [11,12].

The index profile for a standard Luneburg lens of unit radius is given by [13],

(1) $$n(r)=\sqrt{2-r^{2}}$$

This distribution is difficult to implement assuming uniformly sized holes drilled into a host dielectric, because the index goes to unity at the lens boundary. Since the holes have some minimum spacing after which they overlap, an index of unity cannot be achieved since the overall device loses mechanical integrity. Fortunately, the index distribution specified by Equation (1) is not the only solution to the Luneburg lens problem—it is a special case of a more general solution which allows for designs that have non-unity index at the boundary. These refractive Luneburg lenses exhibit some refraction at the lens boundary but still result in perfect focusing. For a lens with a minimum outer index of *n*_0_, the index, *n*, and corresponding radial distance, *r*, are given by the parametric equations [13],

(2) $$\Omega (\alpha )=\frac{2}{\pi }{\int_{\frac{1}{n_{0}}}^{1}\frac{1}{r^{\prime }}\mathrm{atan}\left[\frac{1-\alpha^{2}}{{\left(n_{0}r^{\prime }\right)}^{2}-1}\right]}^{\frac{1}{2}}{\mathrm{dr}}^{\prime }$$

(3) $$n(\alpha )=\{\begin{array}{cc} n_{0}\sqrt{1+{\left(1-\alpha^{2}\right)}^{\frac{1}{2}}}e^{-\Omega (\alpha )} & ,\;\;\;\alpha \leq 1\\ n_{0} & ,\;\;\;\alpha >1 \end{array}$$

(4) $$r(\alpha )=\frac{\alpha }{n(\alpha )}$$

where the parameter *α* goes from zero to *n*_0_.

## 2. Experimental Implementation

In the present refractive Luneburg design, the index value at the lens boundary is determined by the minimum allowed separation of the holes, which is in turn determined by fabrication and mechanical strength constraints.

Once a profile for the untransformed refractive Luneburg lens is obtained, a coordinate transformation is applied that flattens one surface of the lens [5]. Quasi-conformal optimization is applied to the transformation, such that any anisotropy is minimized and can be neglected in the final structure. The degree of flattening of the lens, and thus its field-of-view, is ultimately determined by the dielectric constant of the host dielectric material.

Once a profile for the untransformed lens is obtained, the degree of flattening, and thus the field of view, of the lens is determined by the index of the host dielectric. As the degree of flattening increases, the maximum required index also increases. Since the maximum index cannot be larger than the host dielectric’s index, a second constraint on the index variation of the medium is introduced. The achievable degree of flattening may be increased by introducing multiple material regions so that a low index substrate is used at the outer boundary of the lens and successively higher index materials are used as the prescribed index exceeds those of the outer materials. As is typical for the transformations used to develop transformation optical devices, the transformation applied here extends slightly beyond the boundary of the lens, resulting in some variation of the free space index outside the lens. In addition, the transformation also introduces spatial regions where the refractive index takes values below unity. Values of refractive index less than unity are undesirable, as they imply frequency dispersion and hence introduce bandwidth limitations. Fortunately, approximating these regions by setting their index value to unity has little effect on the focusing behavior of the lens, as will be discussed below.

Once a continuous index profile has been determined, a method for translating that index profile into a distribution of holes must be employed. The resulting distribution should meet several requirements. First, the holes should be uniformly sized, as this greatly simplifies fabrication where drilling or lithography techniques are used. Second, in order for the index to be accurately defined over as small a region as possible and to reduce Rayleigh scattering, the hole distribution should have crystalline symmetry [14]. Third, this crystallinity should be hexagonal, allowing for the maximum range of achievable indices and good isotropy [15].

Several techniques that meet these requirements have been introduced previously. Note that the present flattened Luneburg design is a transformation of an existing gradient index profile, and so the transformation itself cannot be used to arrive at a regular crystalline distribution, as has been done in other transformation optics designs [11]. An alternative technique would be to evenly space holes along lines of constant index, but this approach requires special symmetries to produce good crystallinity, absent from the present design [16]. The algorithm we use treats the holes as a system of interacting particles where the interaction length of each particle is dependent on its position. By allowing the particles to interact, an optimized distribution of holes is achieved with varying spatial density corresponding to the desired index at any location and with local hexagonal symmetry [17].

To investigate the performance of the flattened refractive Luneburg lens, we chose to fabricate and characterize an implementation designed to operate over a broad range of frequencies in the microwave range (at least spanning the 8–12 GHz band measurable in our apparatus). The untransformed lens had a diameter of ten free space wavelengths at our central frequency. The transformation was truncated at the transformed lens boundary. The lens was machined from a slab of Emerson&Cuming ECCOSTOCK HiK polymer-ceramic with index of $\sqrt{6}$ and loss tangent 0.002. The selected design of the refractive Luneburg called for an index of $\sqrt{2}$ at the outer boundary. The host index allows the lens to be compressed by 13.4 percent of the radius, giving a field of view of ±30° and an f-number of 0.433. The lens comprises 7,979 holes. To achieve the aspect ratio required it was necessary for the lens to be drilled halfway through from both sides of the slab using a computer controlled milling machine. Holes of diameter 1/8*^th^* inch were drilled into the ceramic composite material.

**Figure 1.**
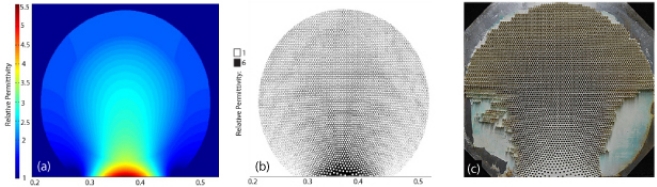
**(a)** The continuous permittivity map for the 2D TE compressed refractive luneburg lens (note the permittivity of two at the outer boundary), **(b)** a distribution of holes with the same effective permittivity and **(c)** the fabricated lens. The hole diameter is 1/8*^th^* inch and the slab thickness is 1*cm*. All distances are in meters.

**Figure 2.**
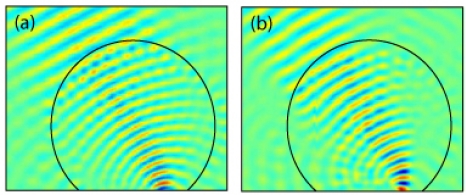
Out of plane electric field for a source located on the flattened focal plane that produces a plane wave propagating at 30 degrees for the **(a)** simulated continuous index lens and **(b)** the measured, fabricated lens. A 10 GHz source was used in both the simulation and the experiment.

Figure 1 shows the optimized relative permittivity distribution for the flattened lens, the hole distribution computed by the particle interaction approach described above, and the final fabricated lens. Plots of the simulated and measured electric field distributions are shown in Figure 2. The field distribution was measured using a 2D electric field mapping apparatus previously reported [18]. For both the simulations and the experimental measurements, a source was placed at the focal plane of the lens, with a roughly collimated beam expected as the output. In the experimental setup, the source consisted of a dielectric rod waveguide with square cross-section coupled to the focal surface of the lens. Simulations were performed using COMSOL Multiphysics, a commercial finite element solver. The agreement between the simulated and measured field patterns was found to be excellent.

## 3. Optical Performance Analysis

**Figure 3.**
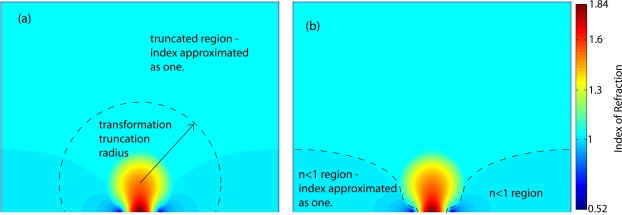
The two approximations made to the transformed Luneburg lens index profile. **(a)** Shows the truncation boundary of the transformation. All indices outside the transformation truncation radius are approximated as one. **(b)** Shows the regions where the transformation index in less than one. These regions are also approximated as one.

In this implementation of the flattened Luneburg, two approximations of the index distribution prescribed by the transformation were made. Because the transformation ideally extends through all space, the free space index outside the untransformed lens is also modified. In order to perfectly preserve the behavior of the Luneburg, the entire transformation should be implemented, but to make a reasonably sized lens the transformation should be truncated close to the transformed lens’s boundary. The deviation from unity index decreases with distance from the lens, but at some point the transformation must be truncated and the index set to one. Thus the first approximation is to set the transformation index to one beyond some radius, as shown in Figure 3(a). Rays incident from free space outside the transformed region will refract at the boundary, slightly distorting their paths. In addition, they will not be guided by the transformation where it has been truncated. For the lens implemented here, the transformation was truncated at the boundary of the transformed lens.

The second approximation has a slightly more severe effect. For regions at the edges of the flattened region of the lens, the transformation indices take values less than one. To avoid using resonant metamaterials, these indices must be approximated as one, as shown in Figure 3(b). This reduces the aperture of the lens because rays that enter the lens at the edge of the aperture, or rays incident from the side of the lens, must pass through these *n* < 1 regions and so are not steered correctly when these regions are removed.

**Figure 4.**
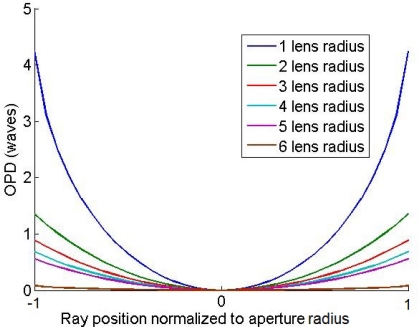
The OPD of rays focused through a transformed Luneburg lens for different size transformation regions. The lens has an aperture size of ten wavelengths and rays are incident along the optical axis of the lens. The legend indicates the radius of a circle, concentric with the lens, beyond which the transformation index is set to one as shown in Figure 3. As the radius of this region is increased, more of the transformation is included and the maximum OPD and corresponding spherical aberration decreases.

The effect of these approximations can be better quantified by performing ray traces through the transformed lens and calculating the optical path difference (OPD) and spot diagrams. Similar ray tracing analysis has been used to compare three dimensional quasi-conformal transformations, which require anisotropic index of refraction, and their isotropic approximations [10]. To simplify the ray-tracing, a traditional non-refractive Luneburg lens was used for the ray traces. For the un-approximated transformed lens, the OPD is zero for all rays and for all angles of incidence. Truncating the transformation region introduces spherical aberration, a fourth order term in the on-axis OPD, as seen in Figure 4. Fortunately at distances just beyond the lens boundary the transformation index is only approximately 1.01 and falls off rapidly, so the amount of spherical aberration introduced by the truncation is small. The maximum amount of spherical aberration occurs when the transformation is truncated at the boundary of the lens.

**Figure 5.**
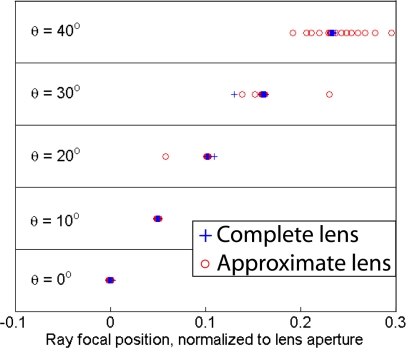
Spot diagrams of 33 rays focused through a complete and an approximate transformed Luneburg lens for different angles of incidence. In the approximated lens, all indices less than one have been approximated as one. The lens has an aperture size of ten wavelengths and angles of incidence are measured from the optical axis. Though the lens has a field of view of ±40°, in the approximated lens rays travel through the approximated *n <* 1 regions for angles greater than 30°

When the *n* < 1 regions of the lens are set to one, rays that pass near the center of the lens are still focused correctly but rays that pass near the edge of the lens aperture are not, reducing the effective aperture of the lens. For rays near normal incidence this approximation has little effect because the only rays that travel through this *n* < 1 region are the rays very close to the edge of the lens aperture. This becomes more of a problem for rays incident far off normal because more rays must travel directly trough the *n* < 1 region outside the lens before entering the lens, as can be seen in Figure 3. The spot diagrams for the un-approximated lens and the *n* > 1 lens are shown in Figure 5. Beyond a certain angle, the spot diagram rapidly spreads out. This is the angle at which rays begin to pass trough the *n* < 1 region outside the lens.

In addition to aberrations introduced by these two approximations, distortion aberrations are also present. This is an interesting effect caused by the transformation itself, and indeed is present in the full-transformation lens as well. In order to preserve orthogonality in the quasi-conformal transformation, the x-coordinates are allowed to ‘slip’ on the flattened boundary, *i.e.*, a Neumann boundary condition is applied to this boundary in the numerical calculation of the transformation [9]. This means that line of constant-x are not uniformly spaced across the flattened boundary, resulting in distortion. This distortion aberration is observed in the OPD plots for off-normal incidence rays. However, because the transformation is known, this distortion can be exactly corrected by applying the inverse transformation to the points on the flattened focal boundary.

## 4. Results and Discussion

**Figure 6.**
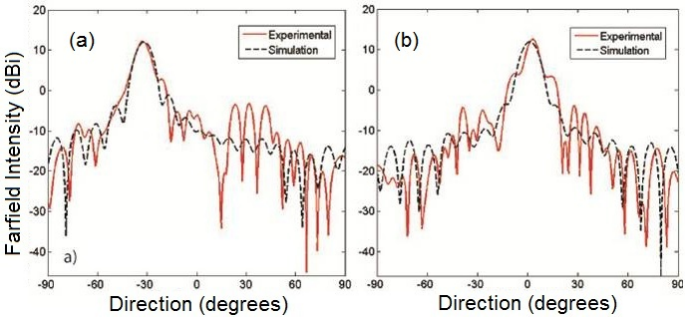
Far-field radiation patterns for the continuous permittivity lens and the experimental lens at **(a)** 30° incidence and **(b)** 0° incidence. Far-fields were calculated from the simulated and experimentally measured electric fields using a 2D Kirchhoff integral.

The performance of the lens was quantified by analyzing the far-field patterns of the lens, shown in Figure 6, using a two dimensional Kirchhoff integral. For all incident angles up to the max of ±30°, the experimental lens exhibits a gain of approximately 16 *dB* and a beam full-width-at-half-max of approximately 8°, comparable to the simulated continuous lens. The disagreement in the side lobes that is seen is due to the inability to achieve permittivities below 1.5 at the lower edges of the lens due to hole percolation. This could be addressed by introducing a lower index host material in these regions.

In conclusion, we designed and fabricated a lens which exhibits focusing over a field of view of ±30° with very little aberration. The design utilizes a refractive Luneburg which has been flattened using a quasi-conformal transformation to produce a flat focusing surface appropriate for planar detector/transmitter arrays. Using the refractive Luneburg as a starting point for our design allowed the lens to be implemented with a semi-crystalline distribution of holes drilled in a dielectric. The design and fabrication approaches are well suited for scaling transformation optical structures to IR and visible wavelengths. The two approximations made in this implementation—the approximation of indices outside the lens and indices less than one as one—have been investigated using ray tracing and were found to introduce only small aberrations.
